# Specialized post-inpatient psychotherapy for sustained recovery in anorexia nervosa via videoconference – study protocol of the randomized controlled SUSTAIN trial

**DOI:** 10.1186/s40337-021-00416-6

**Published:** 2021-05-19

**Authors:** Katrin Elisabeth Giel, Peter Martus, Kathrin Schag, Stephan Herpertz, Tobias Hofmann, Antonius Schneider, Martin Teufel, Ulrich Voderholzer, Jörn von Wietersheim, Beate Wild, Almut Zeeck, Wolfgang Bethge, Ulrike Schmidt, Stephan Zipfel, Florian Junne

**Affiliations:** 1 Department of Psychosomatic Medicine und Psychotherapy, Medical University Hospital Tübingen, Eberhard Karls University Tübingen, Osianderstr. 5, 72076 Tübingen, Germany; 2 Competence Center for Eating Disorders, Tübingen, Germany; 3 Institute for Clinical Epidemiology and Applied Biostatistics, Medical Faculty, Eberhard Karls University Tübingen, Tübingen, Germany; 4 Department of Psychosomatic Medicine and Psychotherapy, LWL-University Hospital Bochum, Ruhr University Bochum, Bochum, Germany; 5 Center for Internal Medicine and Dermatology, Department of Psychosomatic Medicine, Charité - Universitätsmedizin Berlin, corporate member of Freie Universität Berlin, Humboldt-Universität zu Berlin, and Berlin Institute of Health, Berlin, Germany; 6 Institute of General Practice and Health Services Research, TUM School of Medicine, Technical University of Munich, Munich, Germany; 7 Clinic for Psychosomatic Medicine and Psychotherapy, University of Duisburg-Essen, LVR University-Hospital Essen, Essen, Germany; 8 Schoen Clinic Roseneck, Prien am Chiemsee, Germany; 9 Department of Psychiatry and Psychotherapy, University Hospital LMU Munich, Munich, Germany; 10 Department of Psychiatry and Psychotherapy, University Hospital Freiburg, Freiburg, Germany; 11 Department of Psychosomatic Medicine and Psychotherapy, Ulm University Medical Center, Ulm, Germany; 12 Department of General Internal Medicine and Psychosomatics, University Hospital Heidelberg, Heidelberg, Germany; 13 Department of Psychosomatic Medicine und Psychotherapy, Center for Mental Health, Faculty of Medicine, University of Freiburg, Freiburg, Germany; 14 Center for Clinical Trials (ZKS Tübingen), Medical Faculty Tübingen, Tübingen, Germany; 15 Section of Eating Disorders, Department of Psychological Medicine, Institute of Psychiatry, Psychology & Neuroscience, King’s College London, London, UK; 16 Department of Psychosomatic Medicine and Psychotherapy, University Hospital Magdeburg, Otto von Guericke University Magdeburg, Magdeburg, Germany

**Keywords:** Aftercare, Anorexia nervosa, Eating disorder, Inpatient, Psychotherapy, RCT, Recovery, Relapse, Treatment, Videoconference

## Abstract

**Background:**

A major barrier to long-term recovery from anorexia nervosa (AN) are early and frequent relapses after inpatient treatment. There is an urgent need for enhanced continuity of specialized care involving effective aftercare interventions and relapse prevention strategies in order to improve the long-term outcome for patients with AN.

**Methods:**

SUSTAIN is a multi-center, prospective, randomized-controlled trial investigating the efficacy of a novel post-inpatient aftercare intervention for patients with AN as compared to optimized treatment-as-usual (TAU-O). The SUSTAIN aftercare intervention is based on the cognitive-interpersonal maintenance model of AN and specifically tailored to achieve sustained recovery in AN following inpatient treatment. The SUSTAIN aftercare intervention comprises 20 treatment sessions over eight months and will be predominantly delivered via videoconference to overcome discontinuity of care. TAU-O refers to routine outpatient psychotherapy as generally offered in the German health care system. A total number of 190 patients receiving inpatient or day-hospital treatment for AN will be randomized and assessed over a 14-month period following randomization including a 6 months follow-up. Minimum Body Mass Index (BMI) is 15 kg/m^2^ at trial inclusion. The primary efficacy endpoint is the change in BMI between baseline (T0) and end of treatment (T2) adjusted for baseline BMI. Key secondary outcomes comprise eating disorder and general psychopathology, quality of life, proportion of relapse and of weight restoration, and cost-effectiveness.

**Discussion:**

The results of the present trial will provide evidence if the novel aftercare intervention fosters sustained recovery in patients affected by severe courses of AN.

**Trial registration:**

The SUSTAIN trial was prospectively registered on November 18, 2020, under the registration number DRKS00023372 at the German Clinical Trials Register (https://www.drks.de/drks_web/) which is an acknowledged primary register of the World Health Organization (http://apps.who.int/trialsearch/).

**Protocol version:** 1.2.

## Background

A substantial number of patients diagnosed with anorexia nervosa (AN) suffer from a serious course of this eating disorder (ED) [1] and therefore require day-hospital or inpatient treatment [2–4]. Many patients benefit from intense multi-modal hospital treatment in terms of a more rapid weight restoration as compared to outpatient care [5] and improvements in ED pathology at discharge [6, 7]. Unfortunately, initial treatment success often does not translate into sustained positive outcomes after discharge: Long-term follow-up data from a large sample of former inpatients treated for AN found remission rates as low as between 30 and 40% [8], and the mortality risk of former AN inpatients has been found to be five times higher than in the age- and gender-matched general population [2]. This poor prognosis for patients with AN is associated with high relapse rates. A recent review identified relapse rates of up to 53% in patients treated for AN with a relapse risk particularly high as early as 3 months post-treatment [9], and a recent meta-analysis on studies reporting on relapse in AN as the primary outcome yields an overall relapse rate of 31%, predominantly in the first year after treatment [10]. It should be noted though that there is no consensus definition on relapse or recovery in the field of ED [9–12] which makes quantification of the problem challenging and heterogeneous between studies. A further challenge affecting the field of aftercare in AN lies in the situation that a considerable number of adult patients with AN is not weight-recovered when leaving specialized inpatient or day-hospital treatment, but still to some extent fulfills diagnostic criteria for AN or another ED [7]. Therefore, subsequent outpatient care or forms of community support are recommended for most to bridge the transition home [13] and to further improve symptoms. However, apart from these structural and conceptual corner points, there is broad agreement in the field that relapses pose a major barrier to long-term recovery from AN [1, 9, 10]. Two aspects which contribute to these high relapse rates comprise (a) discontinuity of care emerging directly after inpatient release as well as (b) a lack of effective aftercare or relapse prevention interventions addressing patients’ needs after release from hospital treatment. The effective transition between different treatments is an essential component in preventing relapse [13, 14], yet AN patients and carers identify this as challenging. At the same time, guidelines note that there is little evidence of effective relapse prevention strategies [15]. Hence, the urgent need for improved continuity of care involving effective aftercare interventions and relapse prevention strategies in the management of AN has been consistently highlighted in the field [1, 11, 14].

### Existing knowledge

Previous studies have investigated various approaches to provide aftercare as a post-inpatient treatment for adult AN patients [16–23]. Evidence for the effectiveness of pharmacotherapy is inconclusive [20, 21], and high drop-out rates (> 50%) raise questions as to the acceptability of this treatment [21]. Findings on post-inpatient psychotherapy in adult AN patients are more encouraging as this approach appears to prevent weight loss and lead to fewer relapses in the first year after discharge [16, 17, 19, 22]. Two trials investigating digital guided self-help aftercare approaches for AN [18, 23] show this patient group is highly receptive to technology-enhanced dissemination strategies. We have identified two ongoing trials in the field of aftercare for AN [24, 25], one focusses at strengthening self-management skills in patients and their carers [24] and the other one is based on the above mentioned pilot study [23] testing the efficacy of a guided app-based intervention as add-on to treatment-as-usual (TAU) in a larger sample.

Prior to the present randomized-controlled trial (RCT), we have conducted an uncontrolled phase II pilot study to assess need for, feasibility, acceptability and safety of this treatment approach [26]. Within the pilot study, adult AN patients were offered ten sessions of aftercare via videoconference directly after completion of inpatient or day-hospital treatment [26]. The aftercare intervention was based on the same principles as used in the present trial (see below), which is the cognitive-interpersonal maintenance model of AN by Schmidt & Treasure [27] which informs the Maudsley Model of Anorexia Nervosa Treatment for Adults (MANTRA) [28, 29]. MANTRA is based on principles of cognitive-behavior psychotherapy, entails principles from motivational interviewing and targets four putative maintaining factors, including thinking styles, emotional and social skills, close others’ responses to the illness, and pro-anorexia beliefs [27]. The pilot study confirmed that a high proportion of eligible AN patients was interested in participation and that the novel intervention and dissemination via videoconference is feasible and highly accepted by patients and therapists [26]. Moreover, the intervention proved to be safe even for AN patients with a long-standing illness [26].

### Study aims

Based on (a) the promising evidence for the potential of psychotherapy to improve outcomes after inpatient care in adult AN patients, (b) promising results of our pilot study [26] and (c) the acceptability of digital dissemination strategies, we have designed the present SUSTAIN trial.

The primary aim of SUSTAIN is to investigate the efficacy of a novel post-inpatient psychotherapy specifically tailored to achieve sustained recovery in AN following inpatient or day-hospital treatment which will be predominantly delivered via videoconference to overcome discontinuity of care. The novel SUSTAIN post-inpatient psychotherapy will be compared to optimized treatment-as-usual (TAU-O) [30]. The primary efficacy endpoint is the change in Body Mass Index (BMI; kg/m^2^) between baseline (T0) and end of treatment (T2), which is eight months post-randomization, adjusted for baseline BMI. We expect the specialized post-inpatient treatment to be superior to TAU-O in terms of weight gain (BMI) and reduction of ED pathology.

Key secondary aims of the trial comprise the investigation of the effect of the SUSTAIN post-inpatient psychotherapy on the following secondary outcomes: (a) ED pathology, (b) general psychopathology, (c) quality of life, (d) proportion of patients who relapse, (e) proportion of patients who achieve weight restoration (BMI ≥ 18.5 kg/m^2^), (f) BMI course over five assessment points, (g) time to dropout, (h) motivation to change, (i) frequency and lengths of inpatient/day-hospital treatment(s), (j) course of treatment over five assessment points; (k) therapeutic alliance; (l) cost-effectiveness. A further secondary aim is to assess patients’ and therapists’ subjective evaluation of the novel intervention (i.e. acceptability, satisfaction).

## Methods/design

The present study protocol is reported according to the SPIRIT checklist [31]. According to recommendations by the SPIRIT checklist, we describe the intervention following the TIDieR checklist [32].

### Study design and setting

**Fig. 1:**
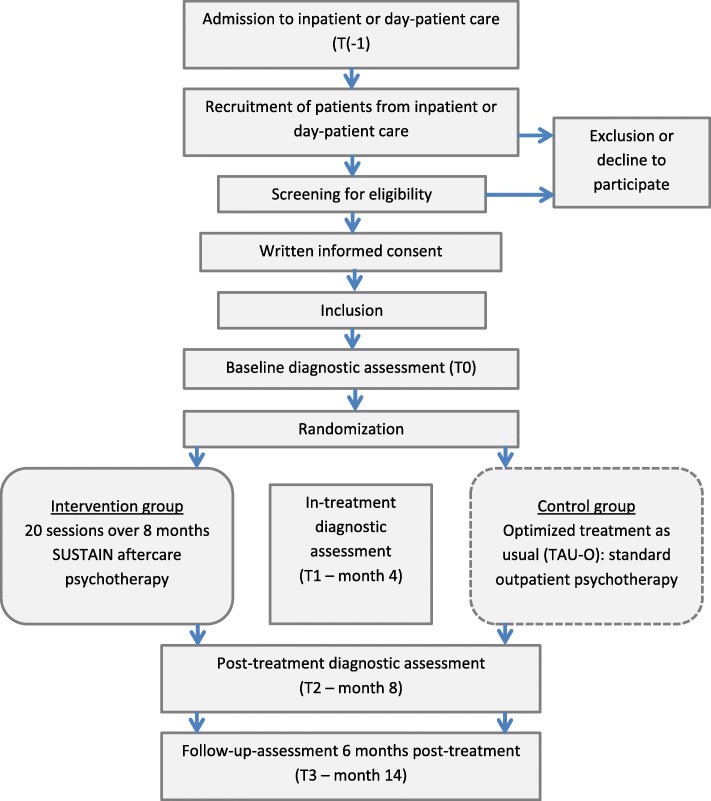
Flow chart of the SUSTAIN trial

SUSTAIN is a multi-center confirmatory superiority RCT with two parallel arms. Patients will be recruited and treated at nine trial sites with specialized wards for inpatient treatment or day-hospital treatment of adults with AN across Germany. In the German health care system, day-hospital treatment programs for patients with EDs are comparably structured and as intensive as inpatient treatment [33]. All AN patients admitted to inpatient or day-hospital treatment at the trial sites will be informed about the SUSTAIN trial and, towards the end of their stay, are invited to participate in the study if they fulfil the inclusion criteria. Figure 1 and Table 1 give an overview on the study process [34]. After providing written informed consent, the baseline assessment takes place in the last seven days before discharge. After completion of the baseline assessment, patients will be randomized at a 1:1 ratio to receive either twenty sessions of the specialized manual-based aftercare intervention SUSTAIN or TAU-O. As Fig. 1 and Table 1 show, the outcomes will be measured at baseline (T0), an intermediate assessment point (T1), at end of treatment (T2) and a 6-month follow-up (T3).

**Table 1:**
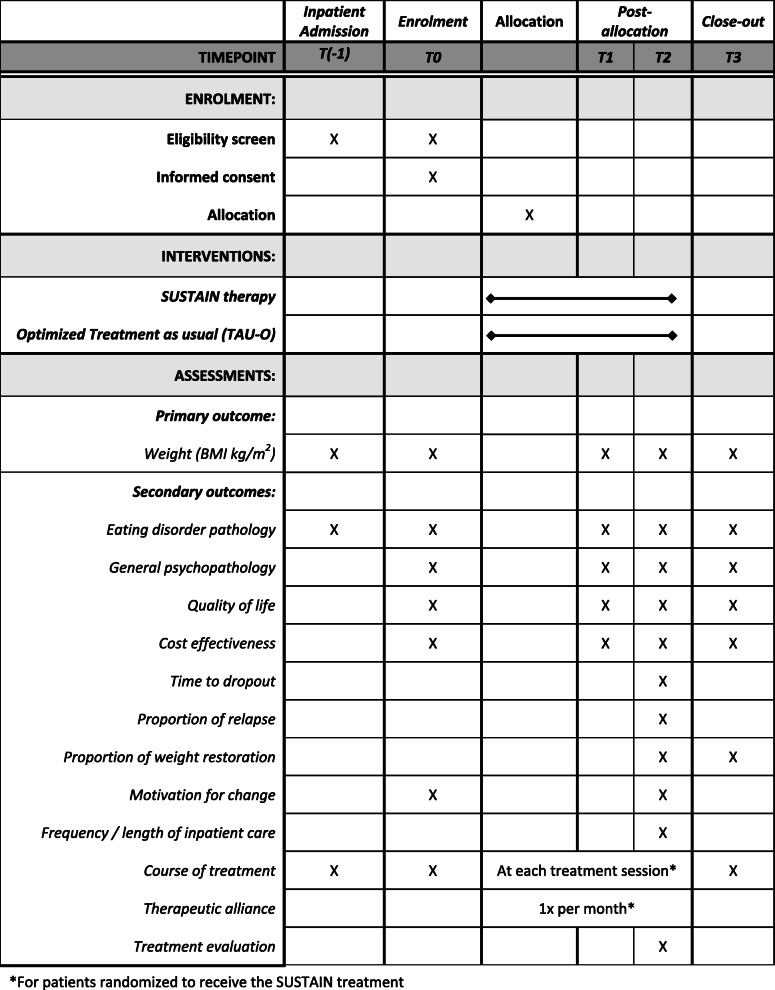
Schedule of enrolment, interventions, and assessments within the SUSTAIN trial according to SPIRIT

### Study participants and eligibility criteria

The study population consists of patients who have been admitted to inpatient or day-hospital care due to a full-syndrome or partial AN according to DSM-5 [35].

#### Inclusion criteria

Patients eligible for the trial must comply with all of the following at randomization:

- age ≥ 18 years
- current admission to the treatment site due to a diagnosis of AN or partial AN at inpatient or day-hospital admission (T(− 1). A full-syndrome AN is diagnosed according to DSM-5 [35] if criteria A, B and C are fulfilled. According DSM-5 [35] recommendations towards partial remission, a partial AN is diagnosed if criterion A is fulfilled and additionally criterion B or C.
- regular completion of inpatient or day-hospital treatment
- minimum weight gain of one BMI point during inpatient or day-hospital treatment
- BMI ≥ 15 kg/m^2^
- written informed consent

#### Exclusion criteria

- acute suicidality
- psychotic disorder lifetime
- manic episode lifetime
- current medium to severe substance use disorder
- severe instable medical problems which require immediate inpatient treatment
- participation in other interventional studies, treatments or therapeutic living community (patients randomized to TAU-O may take up treatments after randomization)

### Interventions

Eligible patients will be randomly assigned to receive either the aftercare intervention SUSTAIN or the control condition TAU-O.

#### SUSTAIN treatment

The novel intervention to be assessed in this trial is specialized post-inpatient psychotherapy for sustained recovery in patients with AN delivered via videoconference (SUSTAIN). SUSTAIN is based on cognitive-behavioral principals of psychotherapy and rooted in the MANTRA treatment approach [28, 29] which is derived from the cognitive-interpersonal maintenance model of AN by Schmidt & Treasure [27]. As outlined above, MANTRA targets four putative maintaining factors, including thinking styles, emotional and social skills, close others’ responses to the illness, and pro-anorexia beliefs [27]. KEG and US enhanced and adapted the treatment manual for the present trial for the application as post-inpatient psychotherapy for severely-ill patients with AN in the context of the German health care system. We created this adaption following a stage model of psychotherapy manual development [36]. The enhanced SUSTAIN manual was successfully piloted in a feasibility study [26]. The treatment manual comprises 10 treatment modules developed considering evidence-based risk and protective factors for relapse in AN. The modules cover an introduction to the intervention and the treatment modules, an individual recovery formulation and traffic light protocol to monitor relapse, identification of treatment goals, working with the support of others, motivation, emotional and social skills, thinking styles, working on an identity independent of AN and maintenance of treatment outcomes. Each module focuses on the relation of the respective dimension to barriers and resources for sustained recovery in AN patients. The modules contain dimension-specific work sheets, illustrations and self-monitoring instruments. The intervention also comprises sessions with significant others, i.e. a family member, partner or friend will be integrated at different stages of the SUSTAIN treatment. The manual encompasses a degree of flexibility to tailor the treatment to the individual patient’s needs by choosing to focus on specific modules with less emphasis on others. Continuity of care will be ensured by two aspects: (a) the intervention will be delivered by specifically trained psychological or physician psychotherapists at the trial site where the patient received inpatient or day-hospital care. (b) The intervention is predominantly delivered via videoconference as proven to be feasible and acceptable in our pilot study [26], in order to reduce barriers to receive psychotherapy for patients living in a large catchment area. The intervention comprises 20 sessions of 50 min each over eight months. In the first two months, weekly sessions take place and afterwards are scheduled bi-weekly.

##### Adherence assessments

Intervention adherence and fidelity will be ensured by several aspects: All psychotherapists delivering treatment in the trial are initially trained in a manual training workshop, and there will be refresher workshops during the course of the trial. Experienced senior psychotherapists at each trial site provide regular supervision. Moreover, regular fidelity checks of randomly chosen recorded therapy sessions are conducted by the study coordinating site.

##### Technical aspects

For the conduction of treatment sessions via videoconference, videoconference solutions are used which are in accordance with current data protection legislation and which are approved for health care use in Germany.

#### Control condition

Due to the severity of AN, a placebo or null intervention control group is ethically inacceptable. We chose optimized treatment-as-usual (TAU-O) as the control intervention. TAU-O has been established as a safe control condition within the ANTOP trial [30] which was previously conducted by our group. TAU-O refers to routine psychotherapy as generally offered in the German health care system in accordance with German general psychotherapy guidelines. The trial site staff will support patients who are assigned to the control group regarding further therapy planning to ensure transfer to outpatient psychotherapy practiced in accordance with the general German psychotherapy guidelines. The term “optimized” refers to standards of patient safety and patient pathways which have been established within a RCT. This includes structured monitoring of patients’ physical health by their general practitioner to manage potential deterioration, as well as the study visits at the study centers for TAU-O patients. Care and intervention dosage received in TAU-O will be closely monitored.

#### Concomitant care

To ensure patient safety, regular monthly visits to their general practitioner are part of the study protocol for all study participants. General practitioners will be informed about the study background and are asked to monitor the patient’s body weight and physical health in order to avoid or at least detect potential deterioration early on. Moreover, a brief inpatient admission, as a crisis intervention for up to four weeks, is possible during trial participation as has been used in the ANTOP study [30]. A crisis admission is arranged if a patient has a BMI < 14 kg/m^2^ over two weeks or in case of severe somatic or mental health complications. The outpatient treatment is interrupted during the crisis intervention. The SUSTAIN treatment can be restarted if the crisis intervention results in successful stabilization within a maximum of four weeks. If this is not the case, the aftercare intervention will be terminated. Concurrent use of psychoactive medications is allowed in the trial, with type and dosage of medication assessed throughout.

##### Prohibited concomitant care

During the active treatment phase of eight months, trial participants receiving the SUSTAIN treatment may not receive any other psychotherapy beyond the post-inpatient aftercare intervention and they may not live in a therapeutic community (see also exclusion criteria above).

### Outcomes

Table 1 gives an overview on assessment points and outcome measures.

#### Primary outcome measure

We chose Body Mass Index (BMI) in kg/m^2^ as our primary outcome measure. BMI is of high clinical relevance, as especially early weight loss after inpatient discharge predicts course of AN [37]. Recent large RCTs investigating efficacy of treatments for AN have used BMI as primary outcome [28, 30, 38], including the two most recent trials investigating a post-inpatient psychotherapy [18, 22]. Current treatment guidelines for EDs across different countries and health care systems emphasize that weight gain and reaching a healthy BMI are key goals of AN treatment [14].

BMI will be calculated based on body height measurement and recurrent measures of body weight at the trial site. The primary efficacy endpoint is the change in BMI between baseline (T0) and end of treatment (T2) eight months later adjusted for baseline BMI.

#### Secondary outcome measures

We use validated structured clinical interviews in combination with validated, common self-report instruments to assess the patient perspective on current symptomatology. Clinical diagnosis of EDs and comorbid mental disorders will be derived from standardized structured expert interviews conducted by trained raters which has been recommended as the gold standard [35].

(a) Eating disorder psychopathology: We will use the *Eating Disorder Examination (EDE)* – clinician and self-report version *(EDE-Q)* to assess ED symptoms and to diagnose the ED [39, 40]. The German version of the *Body Image Questionnaire (BIQ-20)* [41] will be used to assess facets of body dissatisfaction and body image disturbance as core feature of AN.

(b) General psychopathology: We will use the *Structured Clinical Interview for DSM-5 (SCID)* to assess current and lifetime DSM-5 Axis I diagnoses of mental disorders [42]. The *Patient Health Questionnaire (PHQ)* will be used to assess symptoms and severity of major Axis I mental disorders, esp. depression and anxiety as frequent comorbidities of AN [43].

(c) Quality of life: We will apply the self-report measure *EQ-5D-5L* [44] as a generic QoL questionnaire and the *Eating Disorder Quality of Life Questionnaire (EDQOL)* [45] as an ED specific QoL measure.

(d) Proportion of relapse: As there is no consensus definition of “relapse” in AN [9, 10], we define “relapse” in line with criteria used by earlier studies in the field investigating a post-inpatient psychotherapy for AN [17, 21]. This includes (a) loss of 50% of BMI initially restored during inpatient treatment for two consecutive weeks, or (b) (re-)occurrence of severe ED pathology requiring more intensive care, or (c) occurrence of severe medical complication as a result of the ED requiring more intensive care.

(e) Proportion of weight restoration will be defined as the proportion of patients who have reached a BMI ≥ 18.5 kg/m^2^ at the end of their post-inpatient treatment in those with BMI ≤ 18.5 at baseline or BMI maintenance or any increase in patients with a BMI > 18.5 at baseline.

(f) BMI course over five assessment points will be modelled over the whole treatment period of each patient, taking into account T(-1) (admission to inpatient/day-hospital care), T0 (end of inpatient/day-hospital care), T1 (interim assessment after 4 months of post-inpatient psychotherapy), T2 (end of specialized post-inpatient psychotherapy), T3 (end of follow-up).

(g) Time to dropout from post-inpatient psychotherapy will be defined based on the number of therapy sessions completed.

(h) Motivation for change will be measured by the self-report instrument *University of Rhode Island Change Assessment – Short (URICA-S)* [46]*,* which assesses the motivational stages according to the Transtheoretical Model (TTM) by Prochaska & DiClemente [47].

(i) Frequency and lengths of inpatient/day-hospital treatment(s) will be derived from the structured questionnaire used for health economic assessment.

(j) Course of treatment over five assessment points will be derived from treatment documentation implemented in the trial assessment.

(k) Therapeutic alliance will be measured by the self-report instrument *Working Alliance Inventory* (WAI-SR) [48].

(l) Health economic dimensions: The aim of the economic evaluation is to analyse the cost-effectiveness of SUSTAIN. The analyses will consider direct costs (resource utilization) and indirect costs (productivity losses). The assessment of resource utilization and productivity losses is based on the “Client Sociodemographic and Service Receipt Inventory” (CSSRI) [49–52]. We additionally assess in more detail health care use of patients randomized to the TAU-O group.

(m) Treatment evaluation will be conducted using a self-developed self-report evaluation sheet which will cover aspects such as subjective need and motivation for aftercare uptake, feasibility and acceptability of the treatment as well as overall satisfaction [26].

### Participant timeline

The individual participant timeline is depicted in Fig. 1. Study duration for each patient comprises 14 months. This includes the period of eight months intervention and a post-intervention follow-up period of six months. Table 1 shows the four assessment time points during the study (T0, T1, T2, T3). Assessment time point T(− 1) is assigned to the time point of initial admission to inpatient or day-hospital care.

### Sample size

The sample size estimation is based on data from our pilot study [26], where we observed a difference of 1.09 BMI points with a standard deviation of 2.0 in the experimental condition. We conservatively assume a BMI increase of 0.1 BMI points in the TAU-O group with an effect size of 0.495, even though in the literature a stable BMI or a slight decrease was observed under comparable conditions in TAU groups [18, 22]. Therefore, 66 evaluable patients are necessary in each study arm (software query 7.0). To adjust for 30% dropouts, we will recruit 95 patients per study arm, i.e. 190 patients in all. We have based our conservative estimation of the dropout rate of 30% on our pilot trial [26] where we observed 25% dropout. Recent RCTs investigating novel treatments for AN report dropout rates ranging from 20 to 30% [22, 30]. Note that the sample size was calculated for a simple t-test of differences, whereas the primary analysis will be an ANCOVA with baseline adjustment. We expect a decrease of the standard error due to this procedure which will more than compensate the loss of one degree of freedom by inclusion of the covariate and the loss of eight degrees of freedom by inclusion of centre as stratification factor.

### Randomization

Eligible patients will be randomized in equal proportions between the aftercare intervention SUSTAIN or TAU-O. Randomization takes place after completion of the baseline assessment (T0) and is performed independently by the Institute for Clinical Epidemiology and Applied Biostatistics, Tübingen, Germany (ICEAB). Randomization is stratified according to trial site. The ICEAB informs the respective trial site investigator about the group assignment.

### Blinding

Our study design does not allow us to implement blinding of therapists and patients. The study personnel performing the data assessment will be blinded.

### Data management and data monitoring

All data is assessed pseudonymized. Independent data management is provided by the Institute for Clinical Epidemiology and Applied Biometry, Tübingen, Germany (ICEAB). We use electronic case report forms provided by the electronic database secuTrial© (www.secutrial.com) which is access controlled, audit-trailed and certified for Good Clinical Practice (GCP-ICH). Data will predominantly be entered electronically by either the study personnel or patients themselves for self-report measures. Some data will be derived from paper-based source data and transferred to the electronic database; fidelity to the source data will be monitored. Data management will also comprise regular plausibility checks of data entries.

The Centre of Clinical Trials (ZKS Tübingen) at the University Hospital Tübingen is responsible for quality assurance in the present trial and performs regular structured data monitoring in accordance with a fixed monitoring manual. The monitoring procedure includes an initiation visit, intermediate monitoring and a close-out visit. The data monitoring focuses on validation of written informed consent, documentation of (severe) adverse events, validation of inclusion and exclusion criteria, data validity of outcome measures with a special focus on source data transfer and documentation of end of treatment and study dropout.

### Statistical methods

This is an efficacy trial with the primary aim to show superiority of a specialized post-inpatient psychotherapy vs. TAU-O. The primary endpoint (BMI) will be analysed using a baseline (= end of inpatient treatment T0) adjusted ANCOVA for BMI measurement at the end of post-inpatient psychotherapy (T2) with study centre as nuisance factor included. The primary analysis population is the intent-to-treat population (ITT) with imputation of missing data for drop-outs. It is expected that endpoints might be obtained from at least a subsample of drop-outs which might improve the accuracy of imputation procedures for subjects with missing outcome data. Secondary analyses include a linear mixed model for overall course of BMI using BMI at T(− 1) and at T0 as covariates and T1, T2, T3 as dependent observations with predefined analysis: interaction of group with contrasts T2-T1, T3-T1, and (T3 + T2)/2-T1 and estimation of adjusted mean and 95% CI for T3-T2, chi-square test and logistic regression for proportion of relapse. The remaining secondary endpoints will be analysed using adequate methods (chi-square test and logistic regression for binary outcomes, t-test and linear models for continuous outcomes). Time to drop-out will be analysed as a secondary endpoint, using Kaplan Meier and Cox proportional hazard regression. Safety will be analysed using tabulations and line listings of adverse events and separately of severe adverse events. Selected results including the primary endpoint will be analysed in the per protocol (PP) population. We will perform moderator analyses using interaction terms of therapy with potential moderator variables. No interim analysis is planned.

### Ethical aspects

The SUSTAIN trial is conducted in accordance with Good Clinical Practice (GCP-ICH guidelines). Ethical approval for conducting the trial has been obtained by each trial site’s ethics committee. All trial participants provide written informed consent prior to inclusion into the study. Patients can withdraw from the trial at any point without any disadvantage. We have established a Data Safety and Monitoring Board (DSMB) consisting of independent internationally renowned experts in the treatment of AN. The DSMB supervises the entire trial, with a specific focus on the occurrence of adverse events, and evaluates conformity of the trial with the study protocol and ethical standards.

## Discussion

The SUSTAIN trial addresses one of the most serious clinical problems in AN treatment namely early relapse following inpatient treatment, contributing to an unfavourable prognosis. In the present trial, we will investigate the efficacy of a novel post-inpatient psychotherapy specifically tailored to achieve sustained recovery in AN following inpatient or day-hospital treatment. To foster continuity of care, this novel treatment will predominantly be delivered via videoconference and compared to optimized treatment-as-usual (TAU-O) as a control condition [30]. The trial will provide evidence as to if the novel aftercare intervention contributes to a more favorable long-term course in patients affected by severe courses of AN.

In addition, we hope to also contribute with this trial to the field in several other ways: As outlined above, the concepts of recovery and relapse in EDs are currently used heterogeneously [9, 11, 12]; and we believe that with our data, we will be able to contribute to the current work and discussion towards an evidence-based consensus definition for recovery (and relapse) in AN. Secondly, we have implemented a participatory approach within the trial, which means that we have established a lived experience council which consists of people suffering from AN, people recovered from AN and carers of patients with AN. The council members accompany the entire trial, for instance, within the scope of regular council meetings and workshops, sharing their perspectives on needs and preferences of people affected by AN. The council gives advice on the conduct of the study and related processes, e.g. patient-friendly summaries and consent sheets, council members have been involved in the revision of the treatment manual, and they will also support in a later stage in patient- and carer-directed dissemination of results. Thirdly, our data will contribute to the evidence base on using technology-enhanced dissemination strategies to improve treatment access and continuity of care for ED patients. A recent systematic review summarizing findings from RCTs using e-Health interventions for ED patients concludes that the evidence is still very limited, with no trial reporting on the use of psychotherapy via videoconference [53].

We also face several challenges within the present trial: Patients with AN are often ambivalent towards treatment, and especially after completion of intensive inpatient treatment, patients could experience a certain tiredness or reluctance to continue with another treatment, which could influence the rate of aftercare uptake or attrition. However, in our feasibility pilot trial, 70% of eligible patients took up the post-inpatient relapse prevention and patients expressed a high subjective need for such interventions [26] which also motivated us to continue with an efficacy trial. As compared to the pilot trial, we have extended the dosage and duration of the aftercare intervention to the standard duration of outpatient treatment, both in the original treatment approach [28] and in the German health care system, and it remains to be evaluated if this results in more sustained outcomes. The trial has started simultaneously with the COVID-19 outbreak in Europe in spring 2020, posing practical and operational challenges for the conduct of a multicenter trial; and the ongoing pandemic with recurrent lockdown circumstances and high demands towards the health care system might influence recruitment. On the other hand, the dissemination of the treatment via videoconference is more timely then ever and enables us to provide a safe therapy environment under pandemic circumstances. Recent data have shown that people with AN are at risk of experiencing deterioration and relapse during the COVID-19 pandemic [54, 55], and the need to implement digital intervention and dissemination strategies to reach out to vulnerable patient groups has been emphasized [55, 56]. Moreover, whilst the COVID-19 crisis might act as a catalyst for the implementation of digitally assisted treatment delivery [57], evidence-based knowledge on the efficacy is nonetheless needed in order to provide safe and optimal digital mental health practices.

To summarize, we hope that the results from the SUSTAIN trial will contribute to the knowledge on effective aftercare interventions following inpatient treatment for adult patients with a severe form of AN and inform therapists on how this patient group can be supported towards sustained recovery.

## Data Availability

Not applicable.
